# Pharmacological and computational evaluation of fig for therapeutic potential in hyperactive gastrointestinal disorders

**DOI:** 10.1186/s12906-019-2759-2

**Published:** 2019-12-03

**Authors:** Muhammad Bilal Riaz, Arif-ullah Khan, Neelam Gul Qazi

**Affiliations:** Riphah Institute of Pharmaceutical Sciences, Riphah International University, Islamabad, Pakistan

**Keywords:** *Ficus palmata*, Anti-diarrheal, Anti-secretory, Anti-spasmodic, Anti-ulcer, Molecular docking

## Abstract

**Background:**

*Ficus palmata* (Fig), are distributed in different parts of the world, and are used in traditional medicine to treat various ailments including inflammation, tumor, epilepsy, jaundice, influenza and bacillary dysentery. The present study aimed to evaluate the antidiarrheal, antisecretary, antispasmodic, antiulcer and anti motility properties of *Ficus palmata.*

**Methods:**

In-vivo, in-vitro and *in-silico* techniques were used to investigate various gastrointestinal effects of *Ficus palmata*. Antidiarrheal, antisecretary, antispasmodic, antiulcer, anti motility and molecular docking were performed using castor oil induced diarrhea and fluid accumulation, isolated tissue preparations, ethanol-HCl induced ulcer assay, charcoal meal transit time and Auto Doc Vina.

**Results:**

*Ficus palmata* crude extract (Fp.Cr) exhibited protection against castor oil-induced diarrhea in mice and dose-dependently inhibited intestinal fluid secretions. Fp.Cr caused relaxation of spontaneous and K^+^ (80 Mm)-induced contractions in isolated rabbit jejunum preparations. It showed protective effect against gastric ulcers induced by ethanol-hydrochloric acid in rats. Fp.Cr reduced distance travelled by charcoal meal in the gastrointestinal transit model in mice. The plant constituents: psoralenoside and bergapten showed high binding affinities (E-value ≥ − 6.5 Kcal/mol) against histaminergic H_1_, calmodulin and voltage gated L-type calcium channels, while showed moderate affinities (E-value ≥7 Kcal/mol) against dopaminergic D_2_, adrenergic α_1,_ muscranic M_3_, mu-opioid, whereas revealed lower affinities (E-value ≥9.5 Kcal/mol) vs. muscranic M_1_, histaminergic H_2_ and H^+^/K^+^ ATPase pump. Germanicol acetate and psoralene exhibited weak affinities against aforementioned targets.

**Conclusion:**

This study reveals that *Ficus palmata* possesses anti-diarrheal, anti-secretory, anti-spasmodic, anti-motility and anti-ulcer activities. The various constituents reveal different binding affinities against target proteins, which mediate the gastrointestinal functions.

## Background

High prevalence of gastrointestinal disorders among Asian population has caused significant suffering and it is generally considered by the health care professionals to be a leading cause for the incidence of a number of other concomitant disease conditions. Evidence based medicine has not yet succeeded in introducing any sufficiently safe and efficacious drug for the cure of gastrointestinal ailments. Most of the available remedies provide only short-term relief accompanied by various undesirable effects. Nevertheless, conventional phytotherapies have proved to be more cost-effective and long-lasting in the treatment of gastric problems [1]. These herbal products have emerged as a remarkable source to contribute to the discovery of a number of lead compounds and contemporary marketed medicines. Around 61% of the total drugs launched globally have been extracted from naturally originated herbal products [2].

In-vivo evaluation of crude plant extracts aids to screen out newer bioactive lead molecules and their further processing for structural exploration can lead to the development of novel therapies. For obtaining desirable therapeutic effects, pure form of bioactive constituents can be formulated into appropriate dosage forms as well as doses and dosing frequency can be decided [3]. Many chronic disorders have been successfully cured by phytotherapies particularly by edible fruits being consumed as functional foods as well as their active constituents. Several previous studies have demonstrated the potential of crude extracts of a number of edible fruits to treat the gastrointestinal tract diseases [4].

*Ficus palmata* commonly known as ‘Fig’ and locally “Injeer” belongs to the family Moraceae that consists of about 800 species [5]. It is found in the Himalayan region, so also named as Wild Himalayan Fig and is mainly the native of Northern areas of Pakistan. Majority of the members of the family are very tall trees, shrubs and sporadically herbs often with milky juice [6]. Variety of *Ficus* species are used in folk medicine as anti-inflammatory, anti-tumor and tonic medicament [7]. Diseases such as epilepsy, jaundice, influenza, whooping cough, tonsillitis, bronchitis, enteritis, bacillary dysentery, toothache and bruises are also reported to be cured by *Ficus* extracts. Antioxidant activity was exhibited by *Ficus palmata* [8]. Various pharmacological activities such as nephroprotective, hepatoprotective and anticoagulant activities are also possessed by this plant [9].

The chemical analysis on genus *Ficus*, reveals the presence of sterols, terpenes, isoflavones, lignans, glycosides [10], coumarins, furanocoumarin and chromone [11]. Phytochemical investigation of the aerial parts of *Ficus palmata* resulted in the isolation of 6 compounds; germanicol acetate, psoralene, bergapten, vanillic acid, psoralenoside and flavone glycoside rutin [10].

In the present study, we report anti-diarrheal, anti-secretary, anti-spasmodic, anti-motility and anti-ulcer effects. Aforementioned ethnomedicinal uses of the plant were validated by using baseline data from traditional uses and previous studies. Molecular docking of its constituents with known structure is done to find out the potential lead molecule responsible for pharmacological effects.

## Methods

### Plant material and extraction

Superior quality of *Ficus palmata* fruit weighing 2 kg were purchased from local market in Feb 2017. Plant was authenticated by a taxonmist Dr. Mushtaq Ahmad, at Department of Plant Sciences, Quaid-i-Azam University, Islamabad. Voucher specimen no. (ISL-B-24) was collected after submitting sample of specimen of these species to the herbarium at same department. The fruit (2 kg) was air-dried, crushed into powdered form and extracted at room temperature with aqueous-methanol (70:30) three times to obtain *Ficus plmata* crude extract (Fp.Cr).

### Chemicals

Atropine sulphate, omeprazole, verapamil, loperamide, acetylcholine, charcoal, methanol and ethanol (Sigma Chemicals Co, St Louis, MO, USA) were used. Castor oil was obtained from KCL Pharma, Karachi, Pakistan.

### Animals

Sprague-Dawley rats (180–220 g), Balb/C mice (25–30 g) and rabbits (1.0–1.2 kg), of either sex were obtained from animal house of the Riphah Institute of Pharmaceutical Sciences (RIPS) Islamabad. The animals were kept in 595 × 380 × 200 mm plastic cages at standard temperature (23–25 °C) and a 12:12 light:dark cycle with lights on at 08:00 and off at 20:00. They were fed with standard animal feed and tap water ad libitum. Animals were fasted before each experiment for 24 h. During housing, animals were monitored twice daily for health status. No adverse events were observed. All the animal experimental protocols were approved by Research and Ethics Committee of RIPS (Ref. no. REC/RIPS/2017/008) which were performed in accordance with the guidelines of “Principles of Laboratory Animal care” [12]. All sections of this report adhere to the Animal Research:Reported of In-vivo Experiments (ARRIVE) Guidelines for reporting animal research. A completed ARRIVE guidelines checklist is included in Checklist S1.

### Castor oil-induced diarrhea

This method was previously reported by Umer et al. [13]. All the test animals were fasted for 24 h prior to commencement of experimentation and were divided in five groups (*n* = 5). The floor of cage was lined with blotting paper in which animals were placed. First group was assigned as negative control group and received normal saline (10 mL/kg) orally, while second group was given with a dose of loperamide hydrochloride (10 mg/kg, p.o.), assigned as positive control. Third, fourth and fifth groups received 50, 100 and 300 mg/kg body weight of the extract orally respectively. After one hour of administration of the respective doses and treatments, all animals received castor oil (10 mL/kg, p.o.). Post treatment evaluation was carried out after waiting 4 h in order to analyze the diarrheal droppings presence, absence of diarrheal droppings was documented as a positive result.

### Assessment of intestinal fluid accumulation

This method was previously described by Teke et al. [14]. To study the intestinal fluid accumulation, enteropooling assay was used. Overnight fasted mice were taken and put into five assigned cages with five mice in each. Group I and II were administered with normal saline (10 mL/kg) and castor oil (10 mL/kg, p.o.) respectively. Extract doses of 50, 100 and 300 mg/kg intraperitoneally were given to Group III, IV and V respectively.

Standard drug atropine at dose 10 mg/kg was given to last group, 1 h prior induction with castor oil (10 mL/kg, p.o.). Mice were sacrificed by cervical dislocation after 30 min, then intestine was removed and weighed. The results were calculated as: (Pi/Pm) × 1000 where Pi is the weight (g) of the intestine and Pm is the weight of the animal. Later the dead animals were disposed off by burial method.

### Isolated tissue preparation

Before experiment rabbits were fasted for 24 h. Jejunal portion was isolated after cervical dislocation of rabbit and washed with Tyrode’s solution. In tissue organ bath containing Tyrode’s solution 2 cm of jejunal segment was suspended. Temperature of bath was kept at (37 °C) and proper aeration of 95% O_2_ and 5% CO_2_ (carbogen) is ensured. 1 g initial load was applied to tissue and allowed to equilibrate for 30 min before the addition of any drug. Following equilibration period, each preparation was then stabilized with sub-maximal concentration of ACh (0.3 μM) at 3 min interval until constant responses were recorded via a force displacement transducer (model FT-03) coupled with bridge 7 amplifier and power Lab 4/25 data acquisition system connected to computer running Lab-Chart 6 software (AD Instrument, Sydney Australia). The % change in the voluntary contractions of jejunum were recorded for Fp.Cr (0.01–3 mg/mL) [15]. Later the dead animals were disposed off by burial method.

### Ethanol-HCl induced ulcer assay

Rats weighing 250–280 g of either sex were distributed in 5 groups (*n* = 5). Group 1 served as a negative control received normal saline 10 mL/kg body weight, group 2 received 20 mg/kg, (p.o.) omeprazole as standard drug, group 3, 4 and 5 received 50, 100 and 300 mg/kg, (p.o.) of Fp.Cr respectively. All the animals were treated with 1 mL/100 g of ethanol-HCl mixture (p.o.) i.e. (0.3 M Hydrochloric acid and ethanol 60%) after 1 h to induce gastric ulcer. Animals were sacrificed via cervical dislocation one hour after administration of ethanol-HCl mixture. The stomach were removed and lesion index was estimated by measuring each lesion in mm along its greater curvature. Each lesion surface area was measured and scoring was done as described previously by [16]. For each stomach lesion, ulcer index was taken as mean ulcer score (US). For each stomach injury sum of the lengths (mm) of all sores was utilized as the ulcer index (UI). The gastro protective assessment was displayed as an inhibition percentage (I %) calculated by the following formula:

I (%) = (USc – USt) 100/USc.

Where USc = ulcer surface area of control and USt = ulcer surface area of test drug group.

Later the dead animals were disposed off by burial method.

### Charcoal meal transit time

Gastrointestinal transit time was estimated utilizing the charcoal meal test [17]. Rats were fasted for 24 h, the test groups received the extract at 50, 100 and 300 mg/kg body weight doses, where as positive control group received atropine sulfate (0.1 mg/kg, i.p.), while the negative control group received normal saline (10 mL/kg, p.o.). 30 mins after all treatments, all the animals were sacrificed by cervical dislocation. The small intestine was excised after which the distance travelled by charcoal meal through the organ was expressed as a percentage of the length of the small intestine according to the following expression.

Intestinal transit (%) = (Distance moved by charcoal meal/ total length of intestine) (cm) × 100.

Later the dead animals were disposed off by burial method.

### Computational studies

**Fig. 1:**
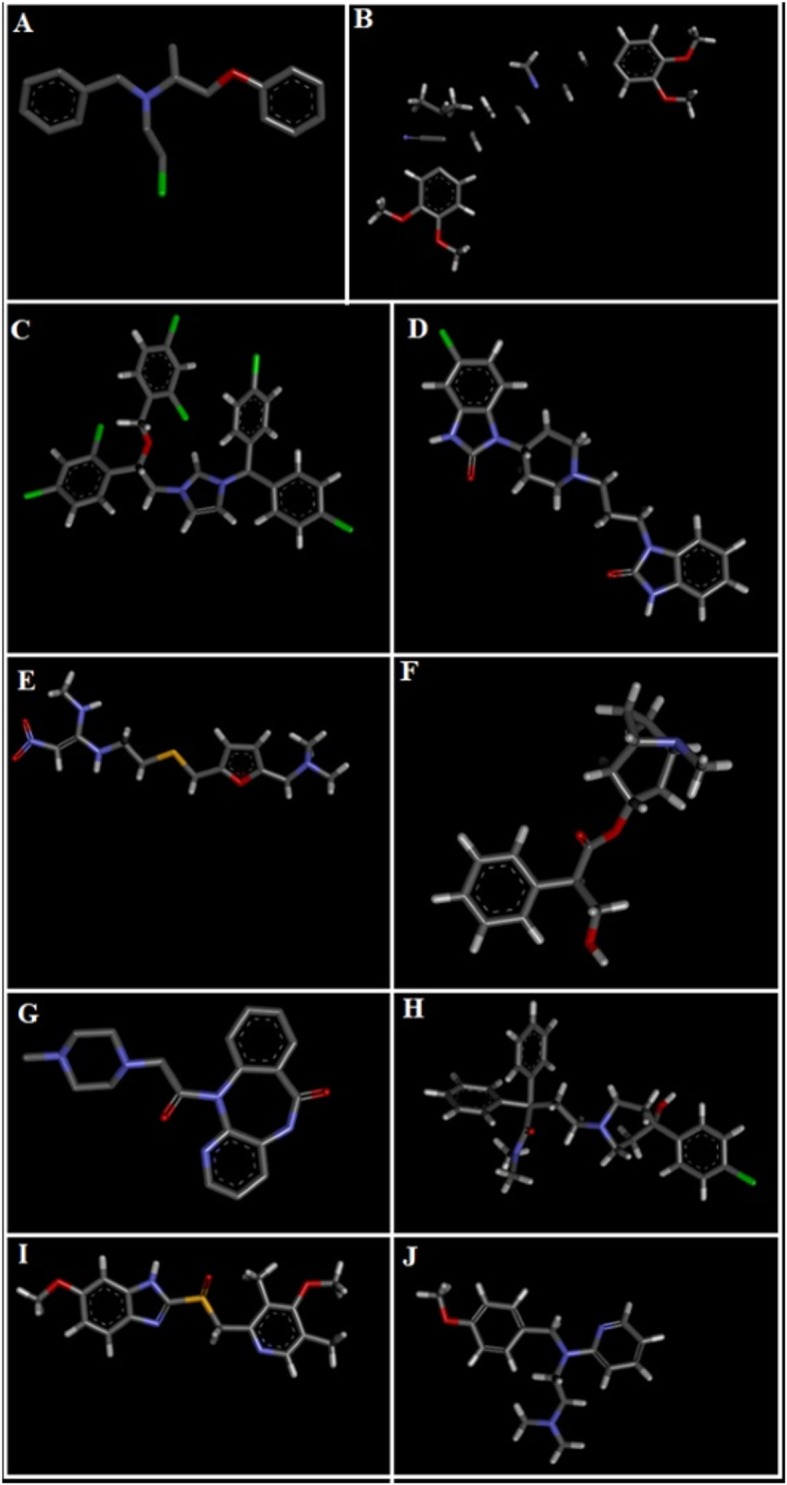
3D-structures of reference drugs: **a** phenoxy benzamine, **b** verapamil, **c** calmozolium, **d** domperidone, **e** ranitidine, **f** piranzapine, **g** atropine, **h** loperamide, **i** omeprazole and (**j**) pyrilimine, drawn through Chem Sketch 2015, 2.5 and saved in PDB format through Biovia Discovery Studio 2016. Atoms are shown by colors; gray color (carbon atoms), white color (hydrogen atoms), red color (oxygen atoms), blue color (nitrogen atoms), dark red (bromine), and yellow color (sulfur atoms)

**Fig. 2:**
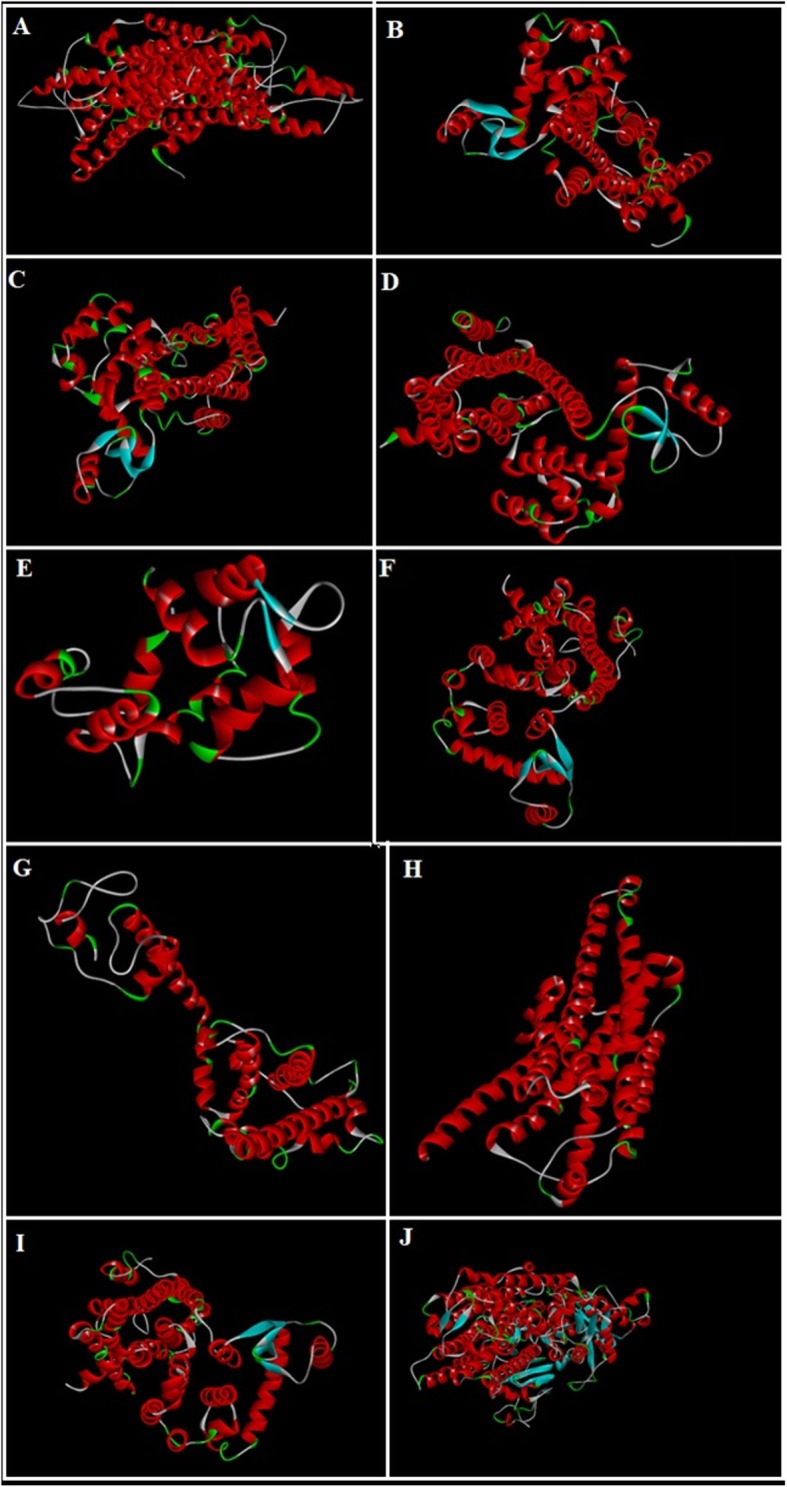
3D-structures of protein targets: **a** adrenergic α_1_ receptor, **b** muscranic M_1_, **c** muscranic M_3_, **d** dopaminergic D_2_, **e** calmodulin, **f** mu-opioid, **g** voltage gated L-Type calcium channel, **h** histaminergic H_1_, **i** histaminergic H_2_, and **(j)** H^+^/K^+^ ATPase pump

3-D structures of the test compounds bergapten, psoralene, psoralenosid and germanicol acetate were constructed by using the software of Gauss View 5.0. 3 dimensional structures of reference drugs were prepared through Discovery Studio Visualizer (2016) as shown in Fig. 1. Through same software, polar hydrogen atoms (H-atoms) were added in it and then saved as PDB file. Reference drugs included phenoxy benzamine, verapamil, calmozoilum, domperidone, ranitidine, piranzapine, atropine, loperamide, omeprazole and pyrilimiine. 3-D structures of selected targets possibly involved in the gut physiology, were retrieved from the website of RCSB protein data bank as represented in Fig. 2. Selected targets included adrenergic α_1_ receptor (PDB ID: 3538), muscranic M_1_ (PDB ID: 5CXV), muscranic M_3_ (PDB 9 ID: 4 U14), dopaminergic D_2_ (PDB ID: 6CM4), calmodulin (PDB ID: 1CTR), mu-opioid (PDB ID: 5CM), voltage gated L-Type calcium channel (PDB ID: 1T3S), histaminergic H_1_ (PDB ID: 3RZE), histaminergic H_2_ (PDB ID: P25021) and H^+^/K^+^ ATPase (PDB ID: 5YLU). Water molecules and ligands were removed along with addition of polar H-atoms by using same software and then saved in PDB format. Autodock Vina which is a geometry based automatic docking tool is used through which molecular docking was performed. A definite value of Root Mean Square of deviation clustering 2 was taken as criteria for elimination of irrelevant findings. Evaluation of docking result was based on atomic energy in Kcal/mol [18]. Assessment of top twenty conformations was performed and only one pose having lowest value of atomic energy in Kcal/mol was further processed for post dock analysis through Discovery Studio Visulizer 2016. Assessment in 2-D design was made to check the most extreme restricting interactions of complex framed amongst amino acid residues and ligands including: valine (VAL), alanine (ALA), proline (PRO), arginine (ARG), threonine (THR), lysine (LYS), proline (PRO), glycine (GLY), glutamine (GLN), asparagine (ASN), cysteine (CYS), methionine (MET), glutamic acid (GLU), histidine (HIS), phenylalanine (PHE), isoleucine (ILE), tyrosine (TYR), serine (SER), threonine (THR), aspartic acid (ASP) and tryptophan (TRP).

### Statistical analysis

Data was expressed as Mean ± SEM (*n* = 5) and median effective concentrations (EC50) having 95% confidence intervals. Statistical analysis of the results was analyzed using one-way ANOVA followed by *post-hoc* Tukey’s test. Chi square test was used in the case of the antidiarrheal data, where *p* < 0.05 was regarded as significant. Non-linear regression using Graph 10 Pad program (GraphPAD, SanDiego, CA-USA) was used to analyze the concentration-response curves.

## Result

### Effect on castor-oil induced diarrhea

Fp.Cr showed a dose-dependent (50–300 mg/kg) protective effect. The saline treated group (negative control) did not show any protection against castor oil-induced diarrhea. Fp.Cr, exhibited 20, 60 and 80% protection from diarrhea at 50, 100 and 300 mg/kg (*p* < 0.05 vs. saline group). Positive control group, loperamide (10 mg/kg) showed 100% protection (*p* < 0.01 vs. saline group) (Table 1).

**Table 1:** Effect of the *Ficus palmata* crude extract (Fp.Cr) and loperamide against castor oil induced diarrhea in mice

Treatment (mg/kg)	No of mice (out of 5) with diarrhea	Protection (%)
Saline (10 mL/kg) + castor oil	5	0
Fp.Cr (50 mg/kg) + castor oil	4	20
Fp.Cr (100 mg/kg) + castor oil	2^*^	60
Fp.Cr (300 mg/kg) + castor oil	1^*^	80
Loperamide (10 mg/kg) + castor oil	0^**^	100

^*^p < 0.05, ^**^p < 0.01 compared to saline group, data analyzed by Chi-squared test

### Effect on intestinal fluid accumulation

**Fig. 3:**
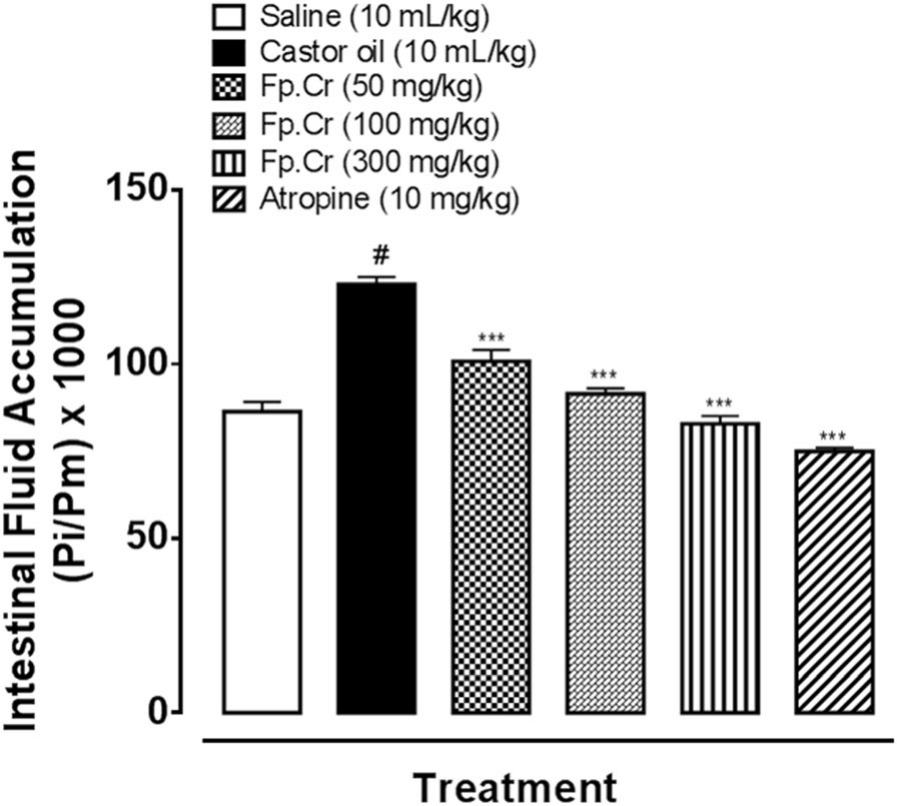
Inhibitory effect of *Ficus palmata* crude extract (Fp.Cr) and atropine on castor oil induced fluid accumulation in mice. Results are expressed as mean ± SEM, *n* = 5. Anti-secretory effect is expressed as Pi/Pm × 1000 (g) where Pi is the weight of the small intestine and Pm is the weight of mouse; ^#^*p* < 0.001 vs. saline group, ^***^p < 0.001 vs. castor oil group, one-way analysis of variance with *post-hoc* Tukey’s test

When tested against castor oil-induced intestinal fluid accumulation in mice, Fp.Cr exhibited a dose-dependent (50–300 mg/kg) anti-secretory effect. In the saline treated group intestinal fluid accumulation was 81.9 ± 0.84 (mean ± SEM, *n* = 5), castor oil-treated group showed 122.5 ± 0.55 (*p* < 0.001 vs. saline group). Fp.Cr at the doses of 50, 100 and 300 mg/kg reduced the castor oil-induced fluid accumulation to 100.30 ± 0.47 (*p* < 0.001 vs. castor oil group), 89.32 ± 0.86 (*p* < 0.001 vs. castor oil group) and 80.98 ± 0.67 (*p* < 0.001 vs. castor oil group) respectively. Atropine at the dose of 10 mg/kg decreased the intestinal fluid accumulation to 74.34 ± 0.69 (*P* < 0.001 vs. castor oil group) as shown in Fig. 3.

### Effect on spontaneous and K^+^ induced contractions

**Fig. 4:**
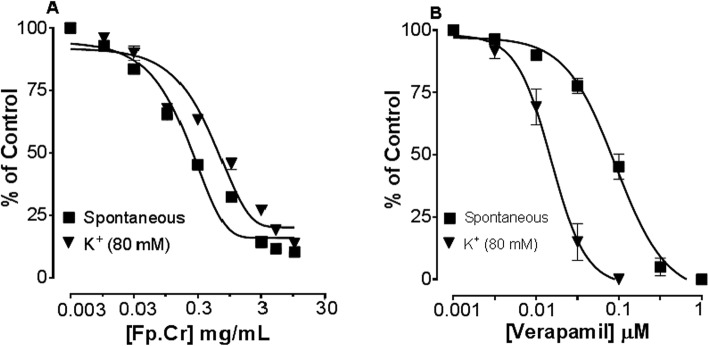
Dose-dependent inhibitory effect on spontaneous and K^+^ (80 mM) induced contractions of (**a**) *Ficus palmata* crude extract (Fp.Cr) and (**b**) verapamil in isolated tissue preparations. Result expressed as mean ± SEM, *n* = 3–5

Figure 6 shows comparative inhibitory effect of the plant extract and verapamil against spontaneous and K^+^ (80 mM)-induced contractions. Fp.Cr was found to be equally effective against spontaneous and K^+^ (80 mM)-induced contractions with EC_50_ values of 0.11 mg/mL (0.08–0.1, *n* = 4) and 0.16 mg/mL (0.09–0.2, n = 4) respectively as shown in Fig. 4a. With EC_50_ value of 0.04 μM (0.03–0.06, n = 4), verapamil was found more potent against K^+^ (80 mM)-induced contractions, as compared to spontaneous contractions [0.12 μM (0.10–0.20, *n* = 3)] as shown in Fig. 4b.

### Effect on ethanol-HCl induced ulcer

**Fig. 5:**
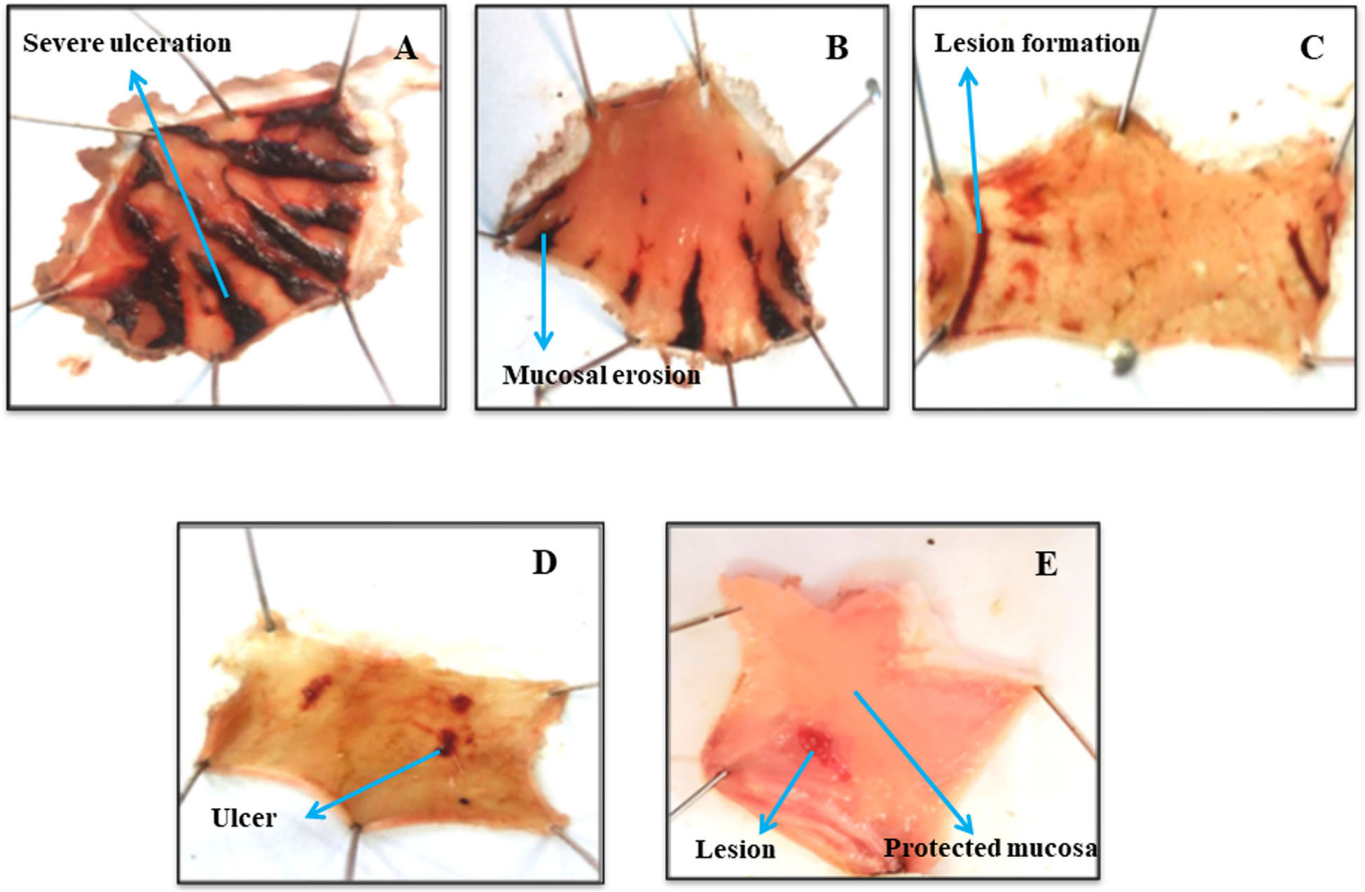
Gross-appearance of gastric mucosa in rat: (**a**) pre-treated with saline, 10 mL/kg (ulcer control). Severe injuries are seen, as ethanol-HCl (1 mL/100 g) produced excessive hemorrhagic necrosis of gastric-mucosa (**b**, **c** & **d**) pretreated with *Ficus palmata* crude extract (Fp.Cr) at doses of 50, 100, 300 mg/kg and (**e**) pretreated with omeprazole 20 mg/kg. The injuries reduce with increase of Fp.Cr doses and omeprazole compare with ulcer-control. At 300 mg/kg, Fp.Cr showed most efficacious gastro protective action

Fp.Cr in dose dependent manner (50–300 mg/kg) exhibited an anti-ulcer effect. Fp.Cr at 50, 100 and 300 mg/kg caused 21.1, 42.2 and 73.3% (*p* < 0.001 vs. saline group) inhibition respectively. Omeprazole (20 mg/kg) showed 88.8% protective effect (Table 2). Macroscopic observation showed the gastric mucosa of rats (Fig. 5).

**Table 2:** Protective effect of *Ficus palmata crude extract* (Fp.Cr) and omeprazole against ethanol-HCl induced gastric ulcers in rats

Treatment	Ulcer Index	% Inhibition
Saline 10 mL/kg + Ethanol-HCl	9.0 ± 0.07	–
Fp.Cr (50 mg/kg) + Ethanol-HCl	7.1 ± 0.20^***^	21.1
Fp.Cr (100 mg/kg) + Ethanol-HCl	5.2 ± 0.14^***^	42.2
Fp.Cr (300 mg/kg) + Ethanol-HCl	2.4 ± 0.14^***^	73.3
Omeprazole (20 mg/kg) + Ethanol-HCl	1 ± 0.11^***^	88.8

^***^p < 0.001 compared to control saline group, one-way analysis of variance, followed by *Post-hoc* Tukey’s test, *n* = 5

### Effect on charcoal meal transit time

Fp.Cr hinders the charcoal meal to travel through the small intestine in a dose dependent manner. The distance travelled by the saline group was 82.29%. Fp.Cr at 50, 100 and 300 mg/kg dose shows inhibition of charcoal meal transit by 50.84, 48.46 and 45.87% respectively (*p* < 0.001 vs. saline group). Atropine (0.1 mg/kg, i.p.) shows inhibitory effect of 44.23% (Table 3).

**Table 3:** Effect of *Ficus palmata* crude extract (Fp.Cr) and atropine on charcoal meal transit time in rats

Treatment (mg/kg)	Mean length of Intestine (cm)	Distance Moved by Charcoal (cm)	Intestinal transit %
Saline (10 mL/kg)	86.00 ± 0.3	70.32 ± 0.6	82.29
Fp.Cr (50 mg/kg)	88.66 ± 0.5	45.08 ± 0.4^***^	50.84
Fp.Cr (100 mg/kg)	86.66 ± 0.4	42.00 ± 0.3^***^	48.46
Fp.Cr (300 mg/kg)	87.00 ± 0.4	40.00 ± 0.5^***^	45.87
Atropine (0.1 mg/kg, i.p.)	90.8 ± 0.6	38.32 ± 0.4^***^	44.23

^***^p < 0.001 compared to control saline group, one-way analysis of variance followed by *Post-hoc* Tukey’s test, n = 5

### Docking evaluation

**Fig. 6:**
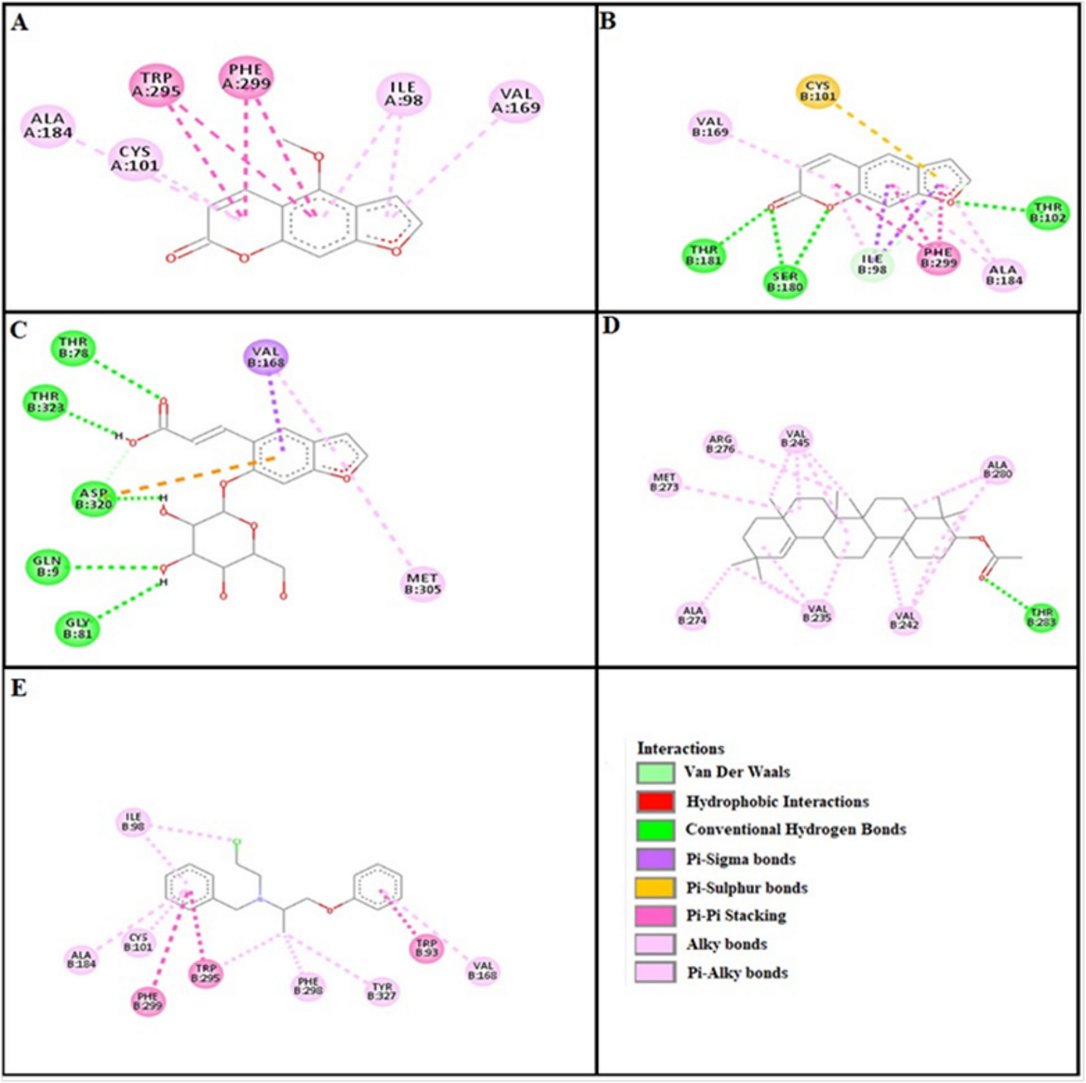
**a**, **b**, **c**, **d** and (**e**) represents interactions of bergapten, psoralene, psoralenoside, germanicol acetate and phenoxy benzamine against target: adrenergic α_1_ receptor respectively, evaluated through Biovia Discovery Studio 2016

**Fig. 7:**
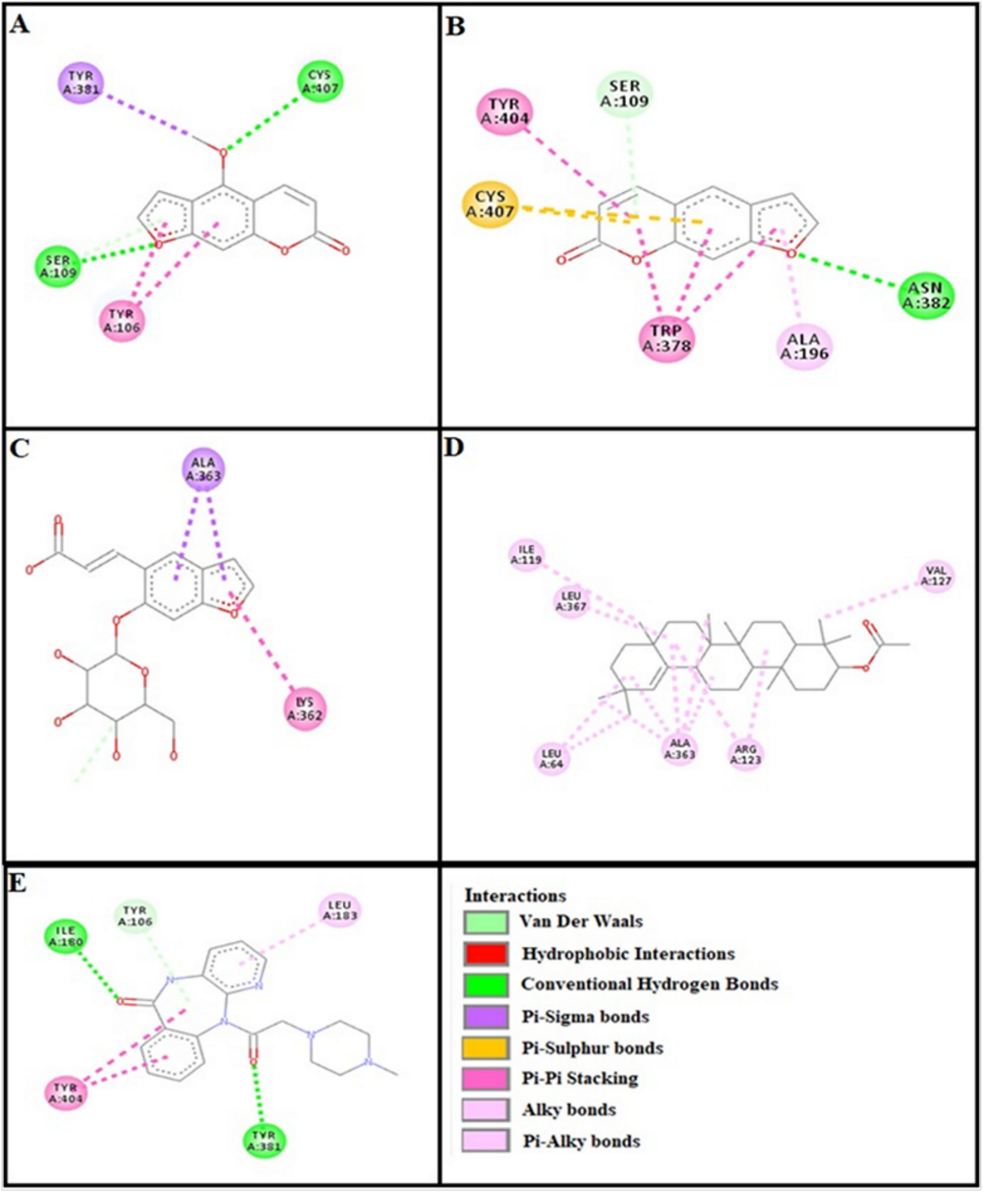
**a**, **b**, **c**, **d** and (**e**) represents interactions of bergapten, psoralene, psoralenoside, germanicol acetate and piranzapine against target: muscranic M_1_ receptor respectively, evaluated through Biovia Discovery Studio 2016

**Fig. 8:**
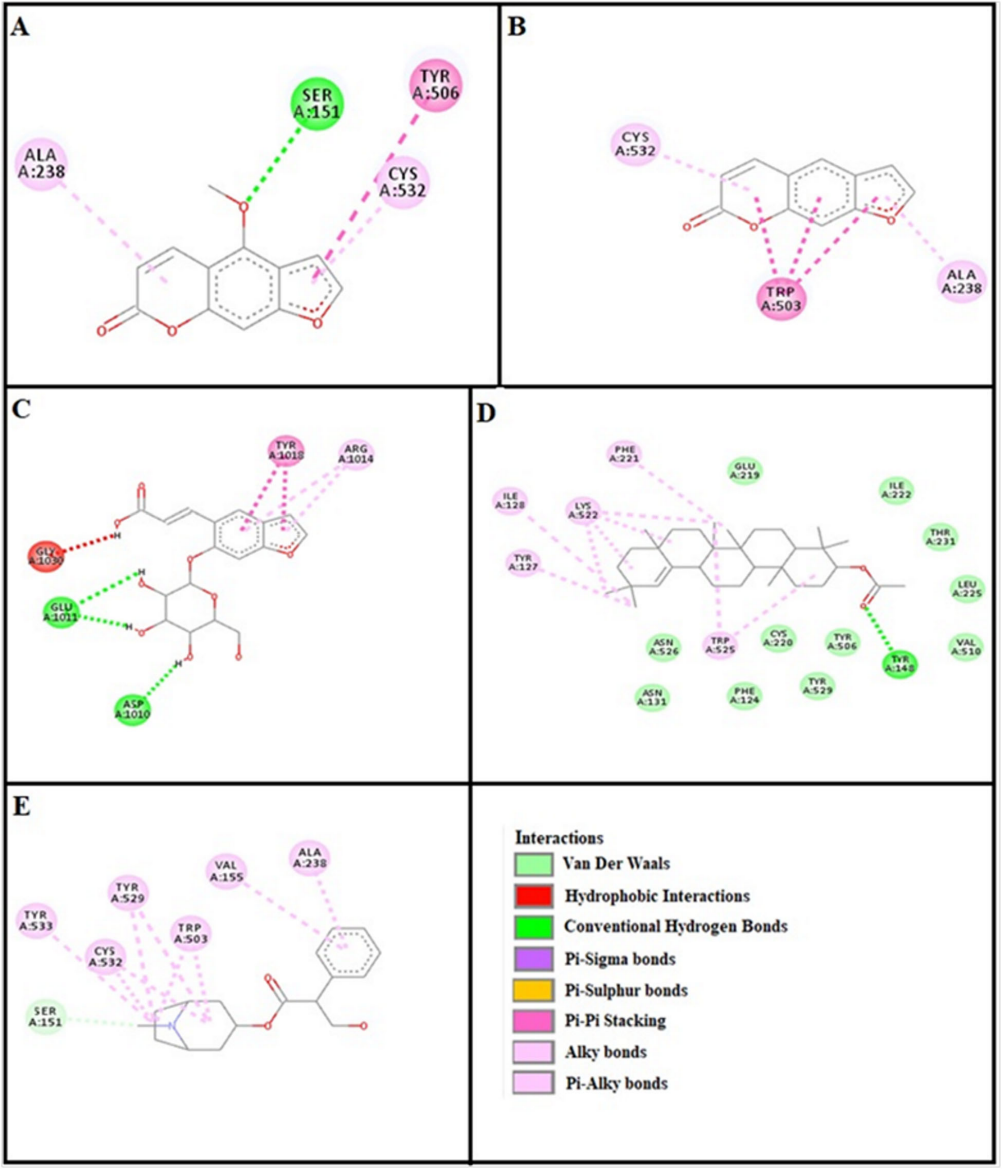
**a**, **b**, **c**, **d** and (**e**) represents interactions of bergapten, psoralene, psoralenoside, germanicol acetate and atropine against target: musranic M_3_ receptor respectively, evaluated through Biovia Discovery Studio 2016

**Fig. 9:**
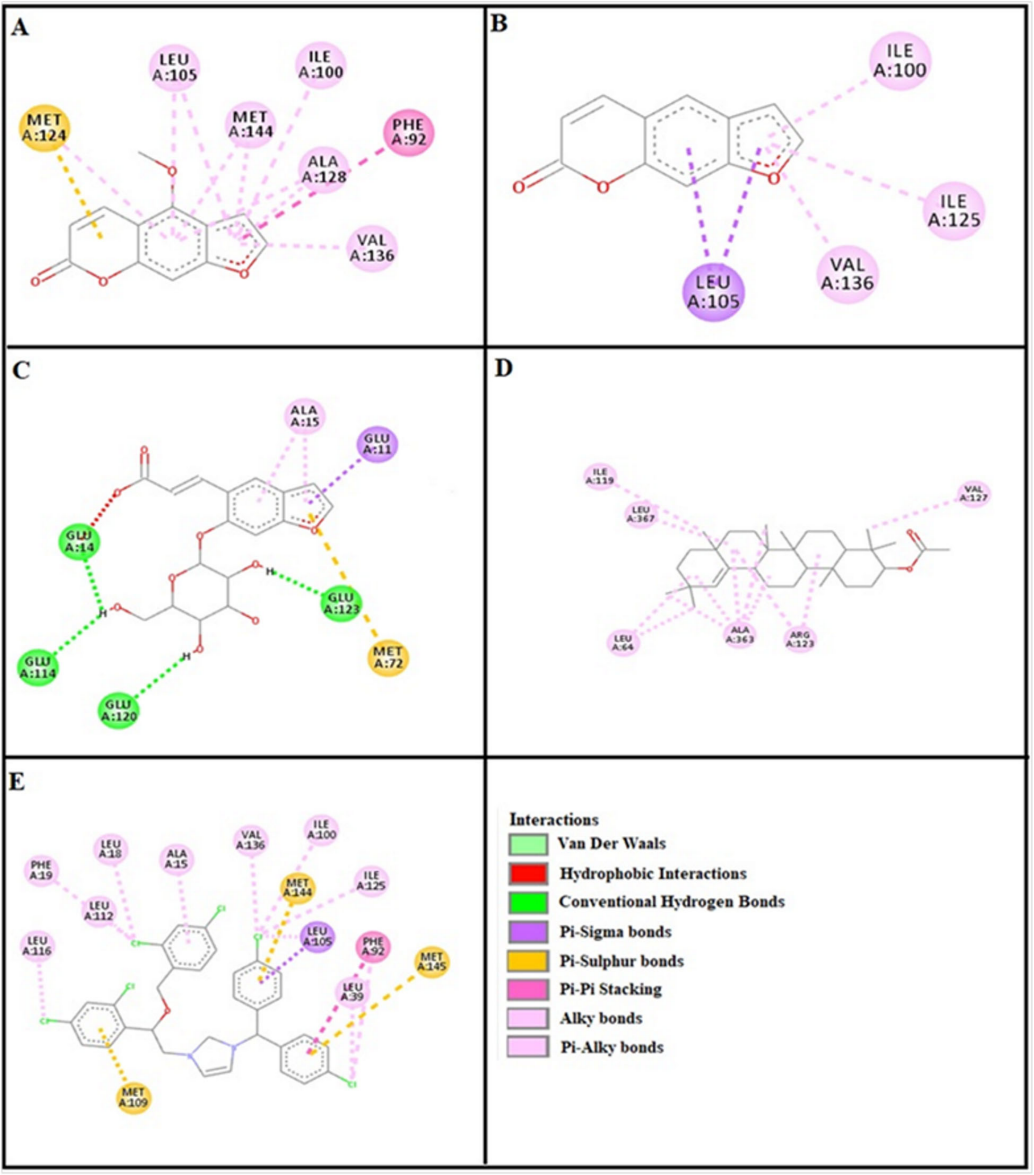
**a**, **b**, **c**, **d** and (**e**) represents interactions of bergapten, psoralene, psoralenoside, germanicol acetate and domperidone against target: dopaminergic D_2_ receptor respectively, evaluated through Biovia Discovery Studio 2016

**Fig. 10:**
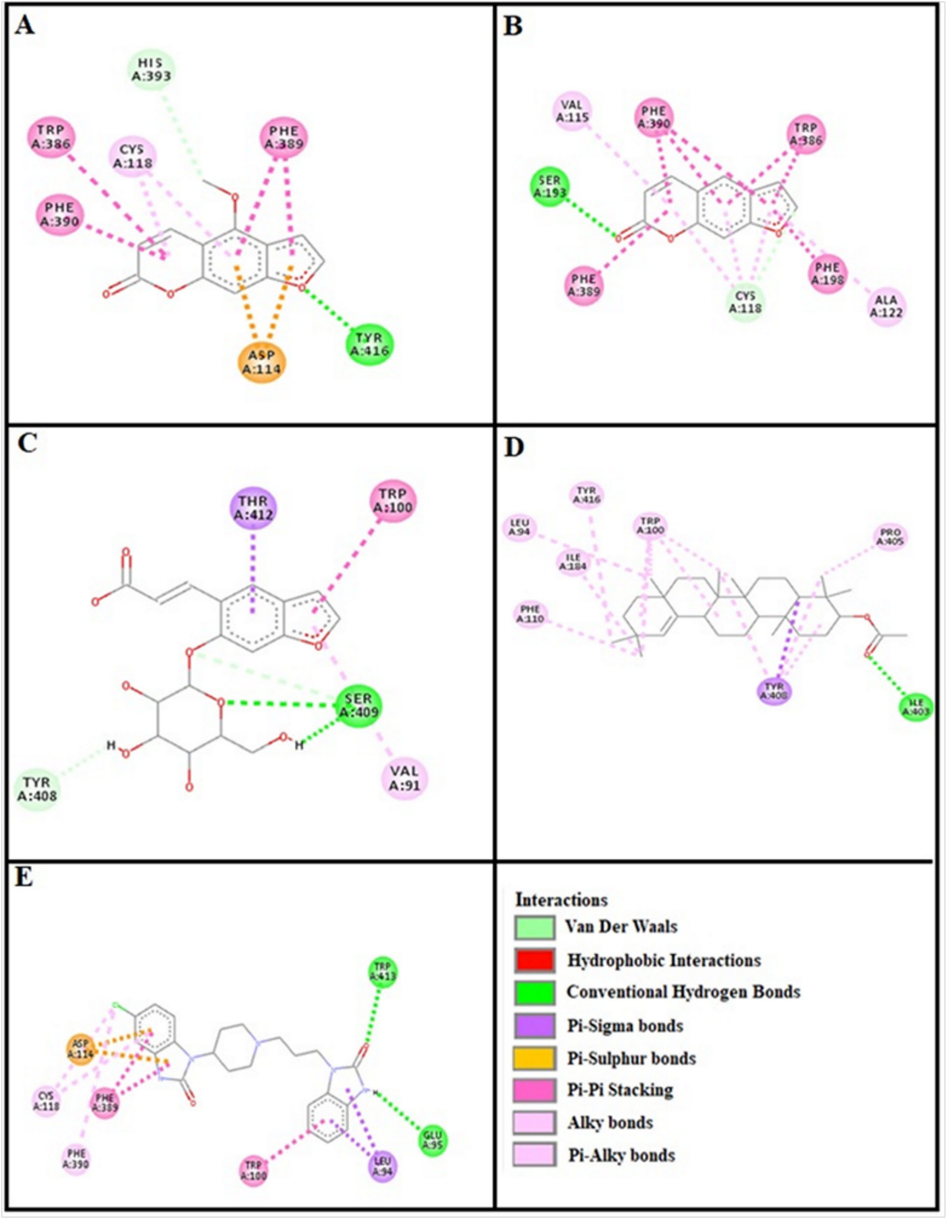
**a**, **b**, **c**, **d** and (**e**) represents interactions bergapten, psoralene, psoralenoside, germanicol acetate and calmozolium against target: calmodulin receptor respectively, evaluated through Biovia Discovery Studio 2016

**Fig. 11:**
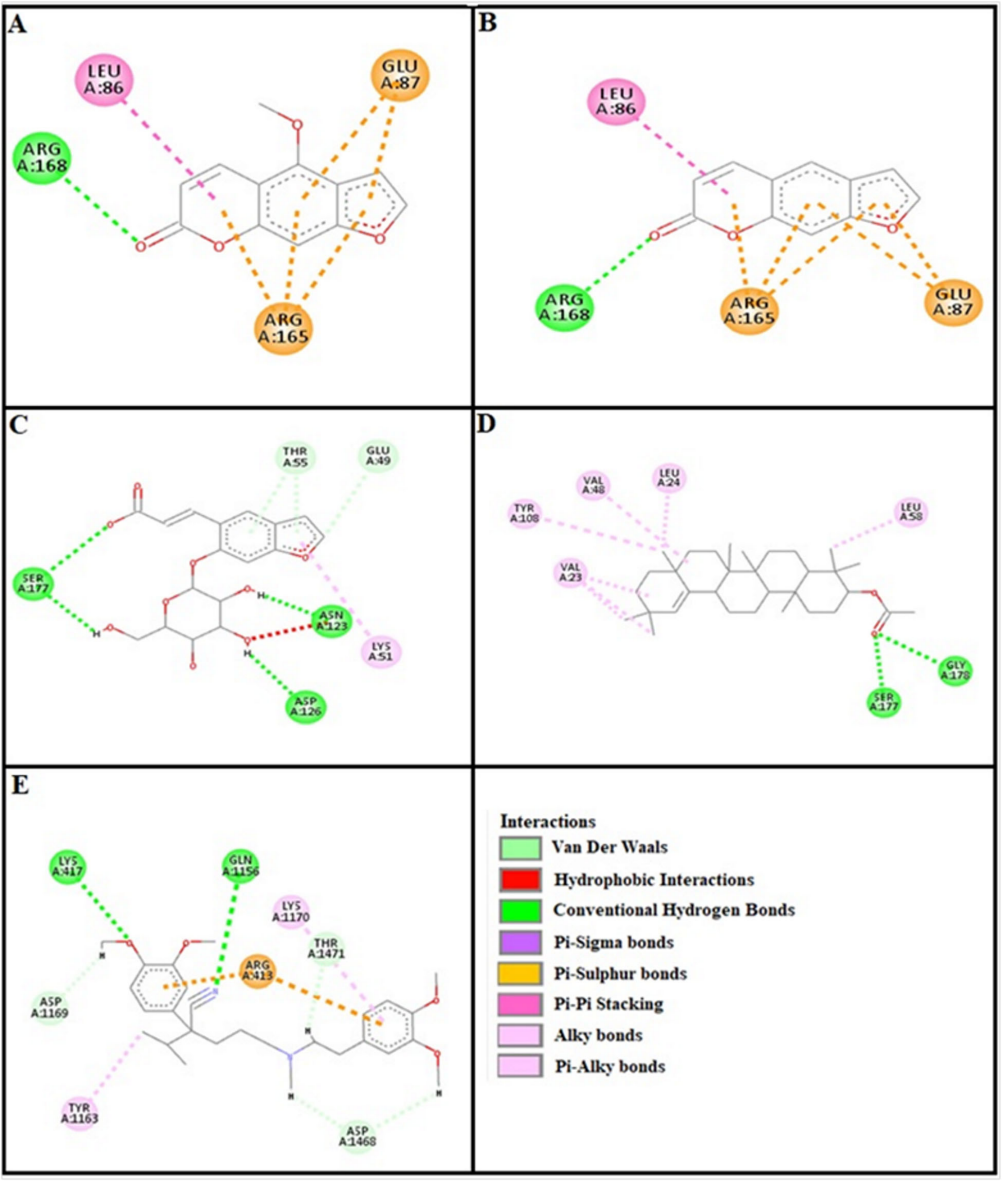
**a**, **b**, **c**, **d** and (**e**) represents interactions of bergapten, psoralene, psoralenoside, germanicol acetate and verapamil against target: voltage gated L-Type calcium channels respectively, evaluated through Biovia Discovery Studio 2016

**Fig. 12:**
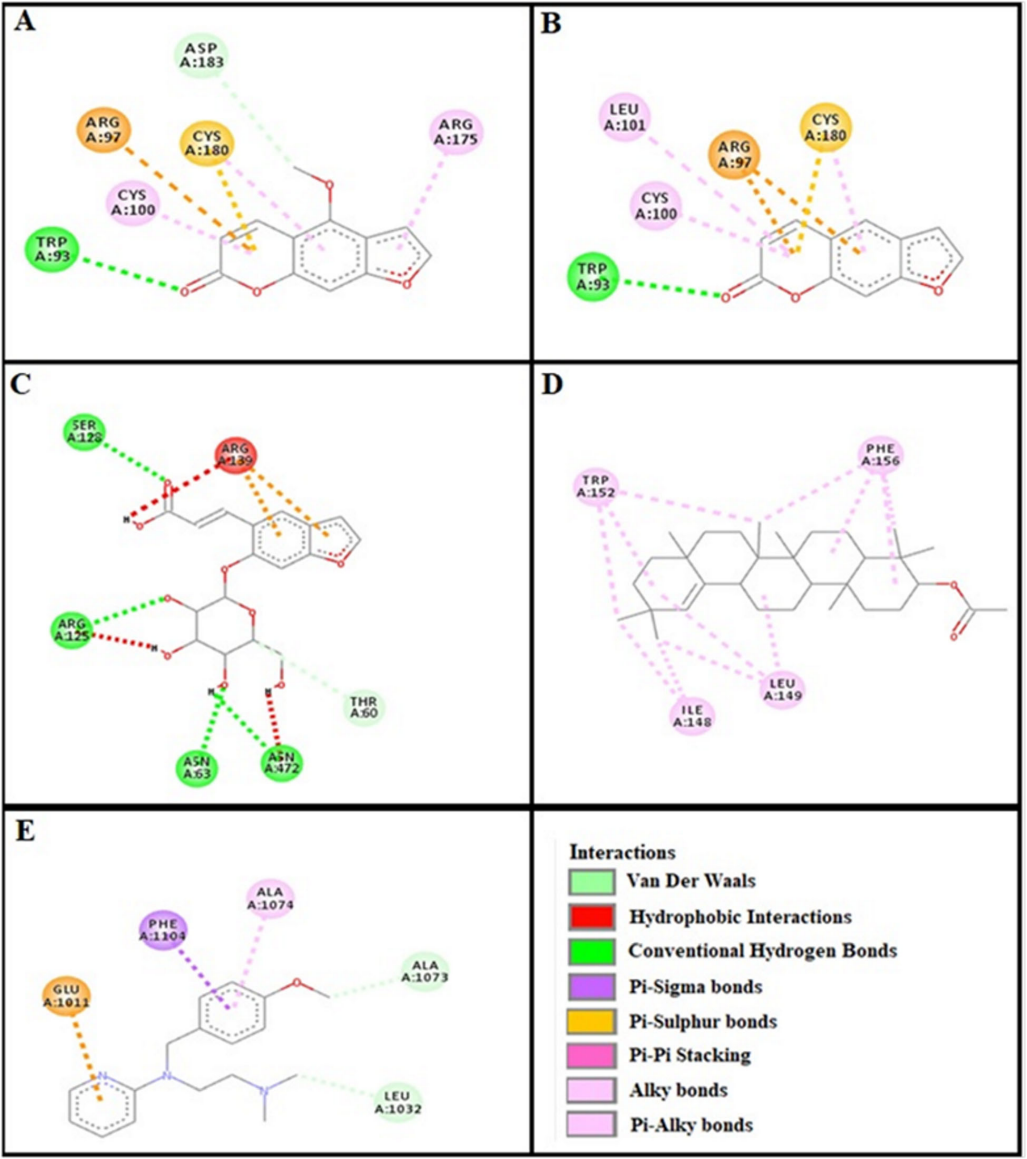
**a**, **b**, **c**, **d** and (**e**) represents interactions of bergapten, psoralene, psoralenoside, germanicol acetate and pyrilimine against target: histaminergic H_1_ receptor respectively, evaluated through Biovia Discovery Studio 2016

**Fig. 13:**
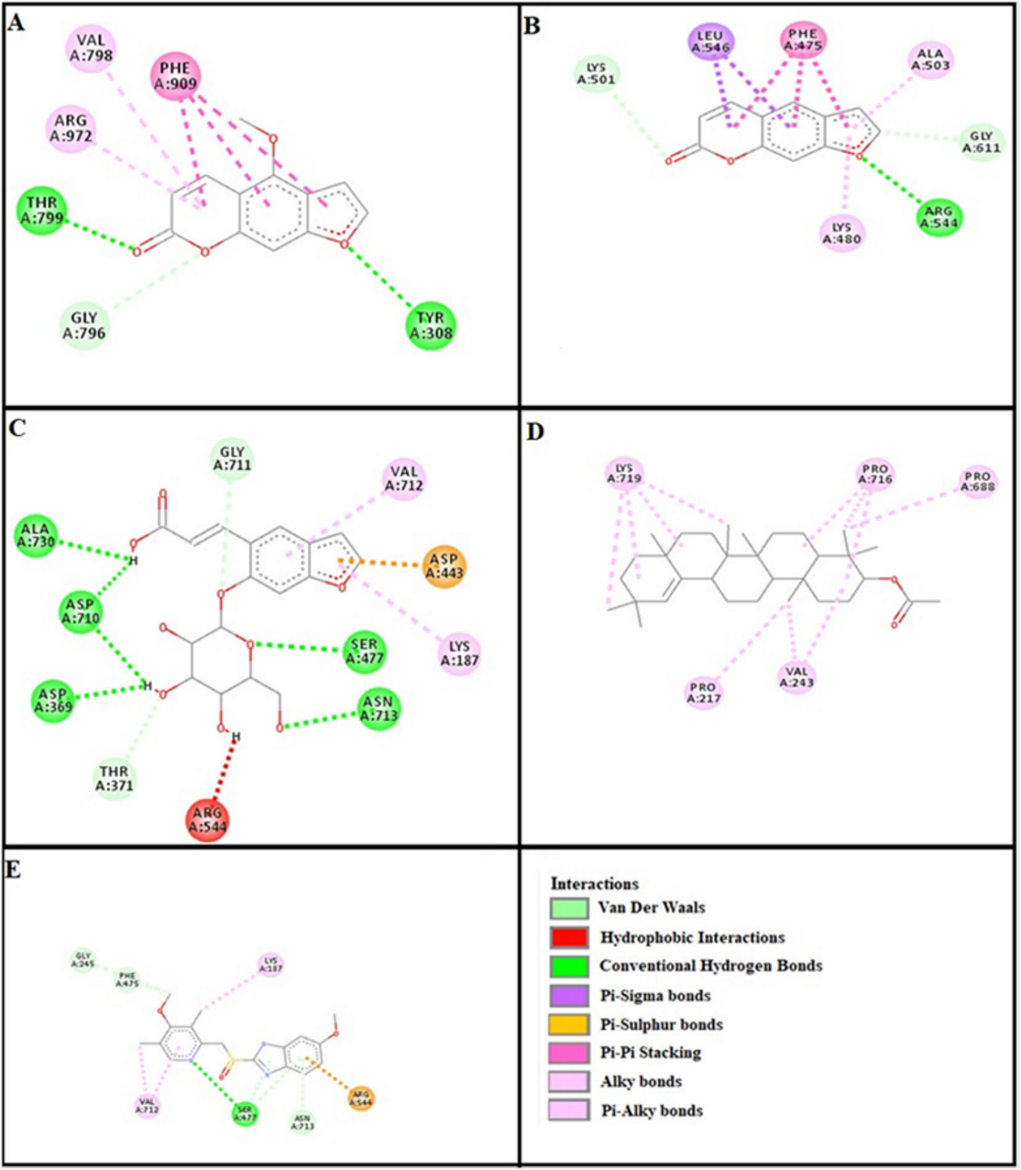
**a**, **b**, **c**, **d** and (**e**) represents interactions of bergapten, psoralene, psoralenoside, germanicol acetate and omeprazole against target: H^+^/K^+^ ATPase receptor respectively, evaluated through Biovia Discovery Studio 2016

**Fig. 14:**
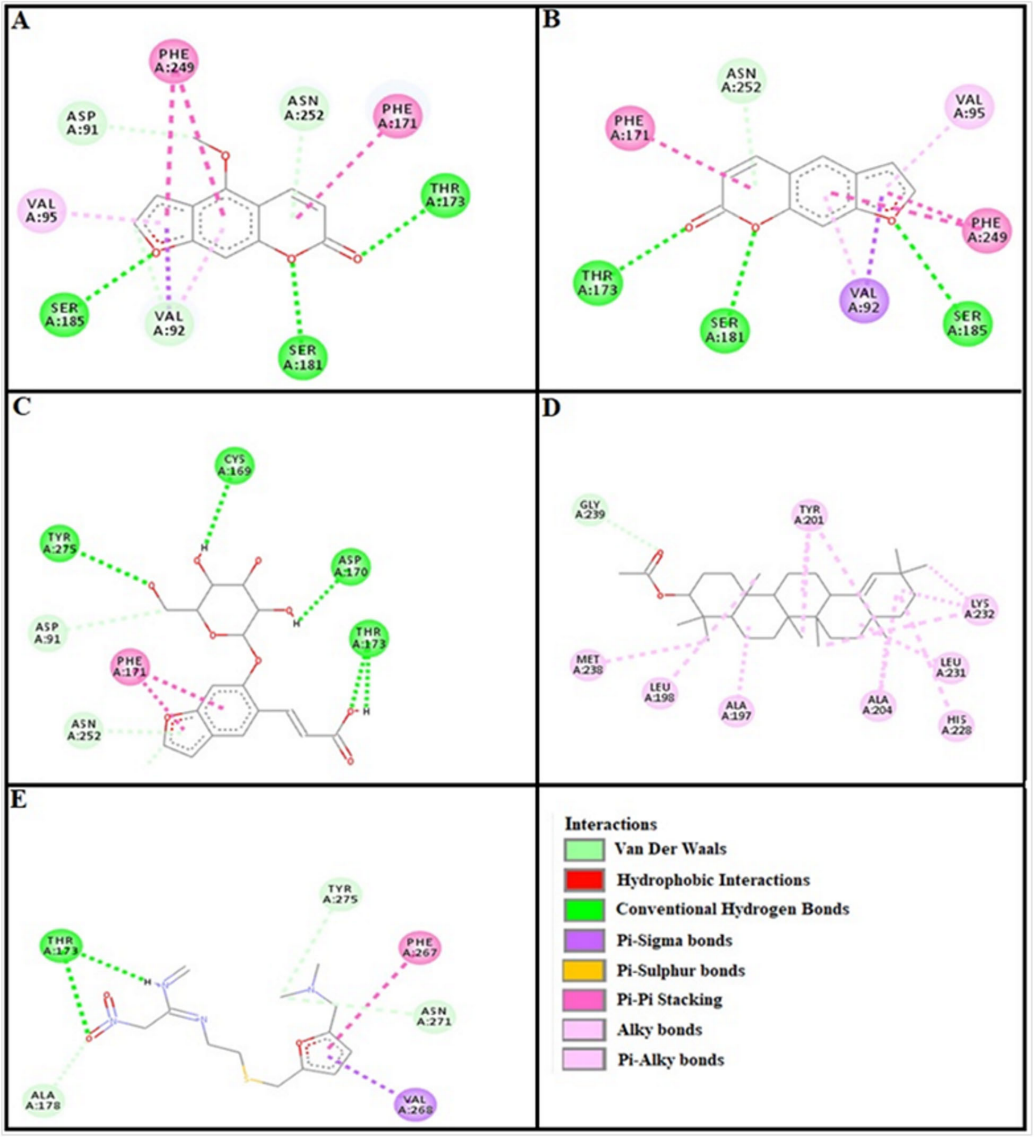
**a**, **b**, **c**, **d** and (**e**) represents interactions of bergapten, psoralene, psoralenoside, germanicol acetate and ranitidine against target: histaminergic H_2_ receptor respectively, evaluated through Biovia Discovery Studio 2016

**Fig. 15:**
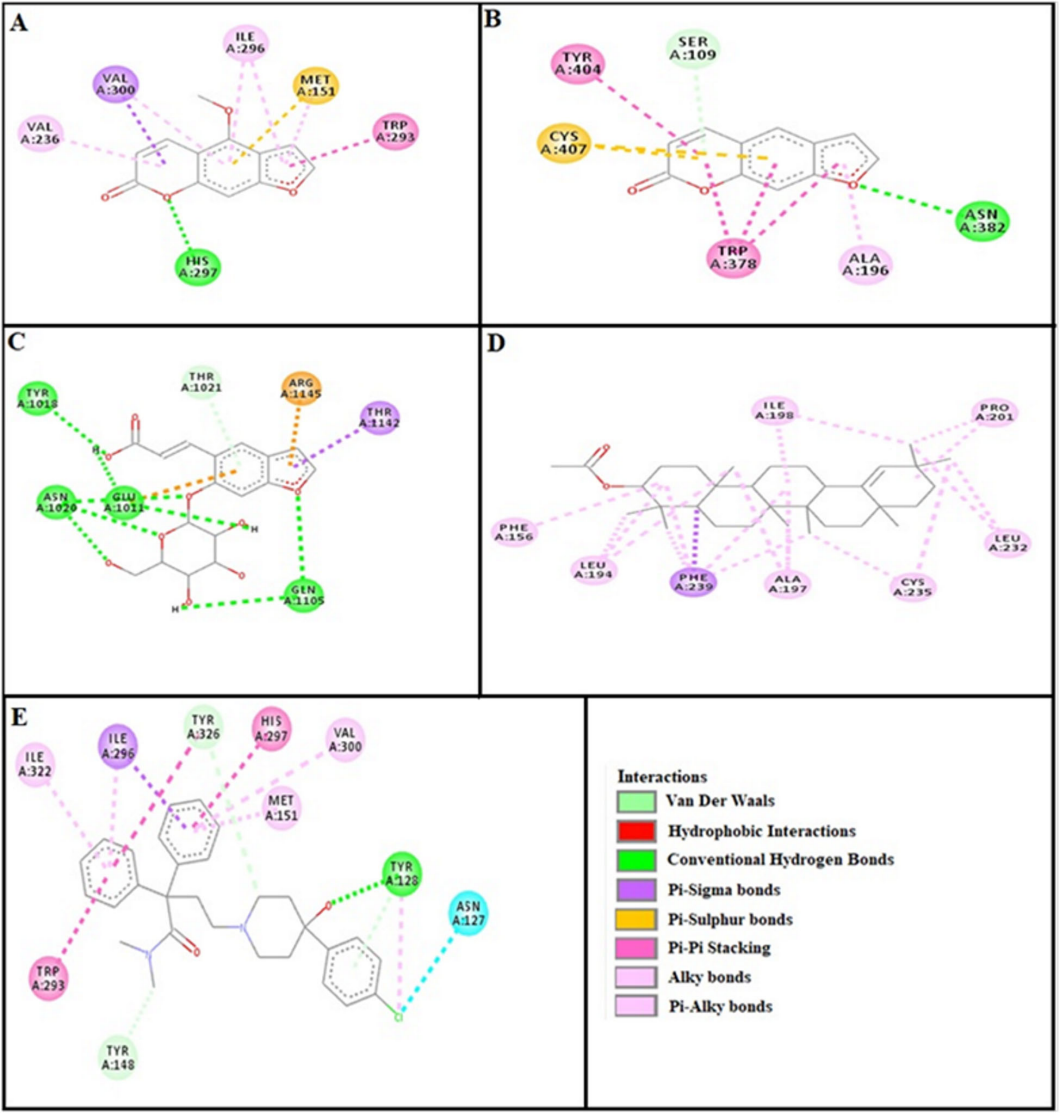
**a**, **b**, **c**, **d** and **e** represents interactions bergapten, psoralene, psoralenoside, germanicol acetate and loperamide against target: mu-opioid receptor respectively, evaluated through Biovia Discovery Studio 2016

Assessment of E-value is an important contributor which helps in docking evaluation. Table 4 shows the values of atomic energy in Kcal/mol for complexes formed between ligand and target receptor. Binding residues involved in the formation of polar hydrogen bonds and total number of hydrogen bonds is expressed in Table 5. Total number of pi-pi bonds and binding residues forming these bonds are represented in Table 6. Other hydrophobic bonding of best models for ligand-target complexes are represented in Table 7. Formation of bonding and interaction by bergapten, psoralene, psoralenoside, germanicol acetate and standard drugs against adrenergic α_1_ receptor, muscarinic M_1_, muscarinic M_3_, dopaminergic D_2_, calmodulin, mu-opioid, voltage gated L-Type calcium channel, histaminergic H_1_, histaminergic H_2_ and H^+^/K^+^ ATPase pump are shown in Figs. 6, 7, 8, 9, 10, 11, 12, 13, 14 and 15 respectively.

**Table 4:** E-values (Kcal/mol) of best docked poses of bergapten, psoralene, psoralenoside, germanicol acetate and standard drugs against targets: adrenergic α_1_ receptor, muscranic M_1_, muscranic M_3_, dopaminergic D_2_, calmodulin, mu-opioid, voltage gated L-Type calcium channel, histaminergic H_1_, histamergic H_2_ and H^+^/K^+^ ATPase pump

Target Proteins	PDB ID	Bargapten	Psoralene	Psoralenoside	Germanicol acetate	Standard drugs
Adrenergic α_1_	3538	−7.7	−8.1	−7.0	10.3	−8.0^A^
Muscranic M_1_	5CXV	−8.1	−8.2	−7.8	− 10.5	− 9.0^B^
Muscranic M_3_	4 U14	−7.5	−7.6	− 7.9	− 9.7	− 8.6^C^
Dopaminergic D_2_	6CM4	− 7.4	− 8.7	−7.3	9.7	−10.6^D^
Calmodulin	1CTR	−5.8	− 5.8	− 6.0	− 9.2	− 8.3^E^
Calcium channel	1T3S	−6.2	− 6.2	− 6.5	− 9.2	− 7.9^F^
Histaminergic H_1_	3RZE	− 6.6	−6.5	−7.0	− 9.0	−5.7^G^
H^+^/K^+^ ATPase	5YLU	−7.4	−8.7	−8.6	− 9.6	− 8.4^H^
Histaminergic H_2_	P25021	−8.2	−8.2	− 8.0	− 9.2	−6.1^I^
Mu Opioid	5C1M	−7.3	− 7.4	− 7.7	− 9.3	− 9.2^**J**^

Standard inhibitors or activator of pathways are: (A) piranzapine, (B) phenoxy benzamine (C) atropine, (D) domperidone, (E) calmozolium, (F) verapamil, (G) pyrilimine, (H) omeprazole, (I) ranitidine and (J) loperamide

**Table 5:** Hydrogen bonds (H-bonds) formed by bergapten, psoralene, psoralenoside, germanicol acetate and standard drugs against targets: adrenergic α_1_ receptor, muscranic M_1_, muscranic M_3_, dopaminergic D_2_, calmodulin, mu-opioid, voltage gated L-Type calcium channel, histaminergic H_1_, histamergic H_2_ and H^+^/K^+^ ATPase pump

Target Proteins	PDB ID	Bargapten		Psoralene		Psoralenoside		Germanicol acetate		Standard drugs	
		H-bonds	Amino Acids	H-bonds	Amino Acids	H-bonds	Amino Acids	H-bonds	Amino Acids	H-bonds	Amino Acids
Adrenergic α_1_	3538	0	–	4	SER 180(2) THR 181 THR 102	5	THR 78 THR 323 ASP 320 GLN 9 GLY 81	1	THR 283	0^A^	–
Muscranic M_1_	5CXV	2	CYS 407 SER 109	1	ASN 382	0	–	0	–	2^B^	ILE 180 TYR 381
Muscranic M_3_	4 U14	1	SER 151	0	–	2	GLU 1011 ASP 1010	1	TYR 148	0^C^	–
Dopaminergic D_2_	6CM4	1	TYR 416	1	SER 193	1	SER 409	1	ILE 403	2^D^	TYR 413 GLU 95
Calmodulin	1CTR	0	–	0	–	3	GLU 114 GLU 14 GLU 127	0	–	0^E^	–
Calcium channel	1T3S	1	ARG 168	1	ARG 168	3	SER 177 ASN 123 ASP 126	2	GLY 178 SER 177	2^F^	GLN 1156 ILE 381
Histaminergic H_1_	3RZE	1	TRP93	1	TRP 93	4	SER 128 ARG 125 ASN 63 ASN 472	0	–	**0**^G^	–
H^+^/K^+^ ATPase	5YLU	2	THR 799 TYR 308	1	ARG 544	5	ALA 730 ASP 710 ASP 369 SER 477 ASN 713	0	–	1^H^	SER 477
Histaminergic H_2_	P25021	3	SER 185 SER 181 THR 173	3	SER 185 SER 181 THR 173	4	TYR 275 CYS 169 ASP 170 THR 173	0	–	2^I^	THR 173
Mu Opioid	5C1M	1	HIS 297	1	ASN 382	3	TYR 1018 ASN 1020 GLU 1011	0	–	1^J^	TYR 128

Standard inhibitors or activators are: (A) piranzapine, (B) phenoxy benzamine, (C) atropine, (D) domperidone, (E) calmozolium, (F) verapamil (G) omeprazole, (I) ranitidine, and (J) loperamide. Amino acids are: ALA, alanine; ARG, arginine; ASN, asparagine; ASP, aspartic acid; CYS, cysteine; GLN, glutamine; GLU, glutamic acid; GLY, glycine; HIS, histidine; ILE, isoleucine; LYS, lysine; MET, methionine; PHE, phenylalanine; PRO, proline; SER, serine; THR, threonine; TRP, tryptophan; TYR, tyrosine and VAL, valine

**Table 6:** Pi-Pi bonds (p-p bonds) formed by Bargapten, Psoralene, Psoralenoside, Germanicol acetate and standard drugs against targets: adrenergic α_1_ receptor, muscranic M_1_, muscranic M_3_, dopaminergic D_2_, calmodulin, mu-opioid, voltage gated L-Type calcium channel, histaminergic H_1_, histaminergic H_2_ and H^+^/K^+^ ATPase pump

Proteins	PDB ID	Bargapten		Psoralene		Psoralenoside		Germanicol acetate		Standard drugs	
		π-π bonds	Amino Acids	π-π bonds	Amino Acids	π-π bonds	Amino Acids	π-π bonds	Amino Acids	π-π bonds	Amino Acids
Adrenergic α_1_	3538	2	TRP 295 PHE 299	1	PHE 299 CYS 181	1	VAL 168	0	–	3^A^	PHE 299 TRP 295 PHE 298
Muscranic M_1_	5CXV	2	TYR 381 TYR 106	3	TYR 404 TRP 378 CYS 407	2	ALA 363 LYS 362	0	–	1^B^	TYR 404
Muscranic M_3_	4 U14	1	TYR 506	1	TRP 503	1	TYR 1018	0	–	0^C^	–
Dopaminergic D_2_	6CM4	4	TRP 386 PHE 386 PHE 390 ASP 114	4	PHE 390 TRP 386 PHE 198 PHE 389	2	THR 412 TRP 100	1	TYR 408	4^D^	ASP 114 PHE 389 LEU 94 TRP 100
Calmodulin	1CTR	2	MET 124 PHE 92	1	LEU 105	2	GLU 11 MET 72	0	–	5^E^	MET 144 MET 145 MET 109 LEU 105 PHE 92
Calcium channel	1T3S	3	LEU 86 GLU 87	2	ARG 65 GLU 87	0	–	0	–	1^F^	ARG 413
Histaminergic H_1_	3RZE	2	ARG 97 CYS 180	2	ARG 97 CYS 180	0	–	0	–	2^G^	PHE 1104 GLU 1011
H^+^/K^+^ ATPase	5YLU	1	PHE 909	1	GLY 611	1	ASP 443	0	–	1^H^	ARG 544
Histaminergic H_2_	P25021	2	PHE 249 PHE 171	3	PHE 171 PHE 249 VAL 92	1	PHE 171	0	–	2^I^	PHE 267 VAL 268
Mu Opioid	5C1M	3	VAL 300 MET 151 TRP 293	3	TYR 494 CYS 407 TRP 378	2	THR 1142 ARG 1145	1	PHE 239	3^J^	ILE 296 HIS 297 TRP 293

Standard inhibitors or activators are: (A) phenoxy benzamine, (B) piranzapine, (C) atropine, (D) domperidone, (E) calmozolium, (F) verapamil, (G) pyrilimine, (H) omeprazole, (I) ranitidine and (J) loperamide. Amino acids are: ALA, alanine; GLN, glutamine; GLY, glycine; HIS, histidine; LYS, lysine; PHE, phenylalanine; SER, Serine; TRP, tryptophan and TYR, tyrosine and VAL, valine

**Table 7:** Hydrophobic interactions formed by bergapten, psoralene, psorelenoside, germanicol acetate and standard drugs against targets: adrenergic α_1_ receptor, muscranic M_1_, muscranic M_3_, dopaminergic D_2_, calmodulin, mu-opioid, voltage gated L-Type calcium channel, histaminergic H_1_, histamergic H_2_ and H^+^/K^+^ ATPase pump

Target proteins	PDB ID	Bargapten	Psoralene	Psoralenoside	Germanicol acetate	Standard drugs
Adrenergicα_1_	3538	ALA 184CYS 101ILE 98 VAL 169	VAL 169ALA 184ILE 98	MET 305	ARG 276VAL 245,242MET 273ALA 280,274	ALA 184LE 98CYS 101VAL 169VAL 168^**A**^
Muscranic M_1_	5CXV	–	ALA 196	–	ILE 119LEU 64,367ALA 363ARG 123	LEU 183TYR 106^**B**^
Muscranic M_3_	4 U14	ALA 238, CYS 532	CYS 532ALA 238	ARG 1014	ILE 128,222TYR 127,506ASN 526,131TRP 525	TYR 533, 529CYS 532TRP 503VAL 155ALA 238^**C**^
Dopaminergic D_2_	6CM4	CYS 118HIS 393	VAL 115CYS 118ALA 122	VAL 91TYR 408	TYR 416LEU 94ILE 184TRP 100	PHE 202ILE 383TYR 213ALA 376GLN 373^**D**^
Calmodulin	1CTR	LEU 105MET 144ILE 100ALA 128VAL 136	ILE 100ILE 125VAL 136	ALA 15	ILE 119LEU 347,64ALA 363VAL 127	PHE 19LEU 116LEU 18ALA 15VAL 136ALA 100^**E**^
Calcium channel	1T3S	–	–	LYS 51THR 55GLU 49	VAL 48LEU 24,58TYR 108	LYS 1170THR 1471ASP 1468TYR 1163^**F**^
Histaminergic H_1_	3RZE	ASP 91VAL 95VAL 92ASN 252	ASN 252VAL 95	ASP 91ASN 252	GLY 239TYR 201MET 238LEU 198,231ALA 197,204	ALA 1074ALA 1073LEU 1032^**G**^
H^+^/K^+^ ATPase	5YLU	VAL 798ARG 972GLY 796	LYS 501LYS 480ALA 503GLY 611	GLY 711VAL 712LYS 187THR 371	LYS 719VAL 243PRO 716, 688	GLY 245PHE 475VAL 712ASN 713LYS 187^**H**^
Histaminergic H_2_	P25021	ASP 91VAL 95VAL 92	ASN 252VAL 95	GLY 239MET 238LEU 198, 231HIS 228ALA 197	ALA 269VAL 72, 268TRP 265	TYR 275ASN 271ALA 178^**I**^
Mu-Opioid	5C1M	ILE 296VAL 236	SER 109ALA 196	ILE 198PHE 156LEU 196ALA 197	ILE 322, 296TRP 318VAL 300HIS 319TYR 128	ILE 296TYR 326VAL 300MET 151ILE 322^**J**^

Standard inhibitors or activators are: (A) phenoxy benzamine, (B) piranzapine, (C) atropine, (D) domperidone, (E) calmozolium, (F) verapamil, (G) pyrilimine, (H) omeprazole, (I) ranitidine, and (J) loperamid.. Amino acids are: ALA, alanine; ARG, arginine; ASN, asparagine; ASP, aspartic acid; CYS, cysteine; GLN, glutamine; GLU, glutamic acid; GLY, glycine; HIS, histidine; ILE, isoleucine; LYS, lysine; MET, methionine; PHE, phenylalanine; PRO, proline; SER, serine; THR, threonine; TRP, tryptophan; TYR, tyrosine and VAL, valine

## Discussion

Based on ethnopharmacological use of *Ficus palmata* in hyperactive gut diseases, such as colic and diarrhea, its extract was evaluated for the possible anti-diarrheal, anti-secretory, charcoal meal gastrointestinal motility and anti-ulcer effects in rodents. Isolated intestinal tissue was used for the elucidation of possible underlying mechanism(s) to rationalize aforementioned ethnomedicinal uses of the plant and it was further supported by virtual screening tool**s.**

Fp.Cr demonstrated an excellent antidiarrheal activity against castor oil induced diarrhea similar to the effect produced by loperamide, a standard drug [13]. Castor oil cause increases in the peristaltic activity and induce permeability changes to electrolytes and water in the mucosal membrane of the small intestine similar to an effect which is associated with prostaglandin release. Ricinoleic acid which is an active metabolite of castor oil is another major factor in causing diarrhea through a series of actions including activation of small intestinal peristaltic activity with reduction of Na^+^/K^+^ ATPase activity. These changes eventually results in disturbance in the intestinal mucosa, electrolyte permeability, hypersecretion of intestinal contents and a slogging of the transport time in the intestine [19]. Thus, a potential agent may exhibit its anti-diarrheal activity by these mechanisms. Intracellular Ca^2+^ levels had a huge impact on secretary functions of the gastrointestinal organs which lead towards consequences such as discharge of gastric acids and intestinal fluid release. This effect might be affected by some drugs that hinder calcium influx [20]. Fp.Cr shows protection against castor oil induced intestinal fluid secretions in mice. The anti-diarrheal and anti-secretory activities of Fp.Cr might be because of gastrointestinal relaxant component(s) present in the Fp.Cr [10].

Dose-dependent inhibition of spontaneous contractions in isolated rabbit jejunum preparations were exhibited by Fp.Cr, thus showing antispasmodic action. High K^+^ (80 Mm), as KCl, was used to depolarize the tissue to analyze whether the spasmolytic activity of this extract was mediated through calcium channel blockade or some other mechanism. High K^+^ (80 Mm) is known to cause smooth muscle contractions with opening of voltage gated L-type calcium channels, thus permitting the inflow of extracellular calcium causing contractility and the substance causing this inhibition of high K^+^-induced contractions is considered as calcium channel blocker [15]. Verapamil, a specific calcium antagonist have inhibitory effect against K^+^-induced contractions. Against spontaneous and K^+^-induced contractions Fp.Cr produces inhibitory pattern just like verapamil.

Gastric ulcer is the result of an imbalance between aggressive and defensive factors of the gastric mucosa which results in rupturing of mucosal protection and expose gastric lining to gastric acids. To explore the anti-ulcer effect of Fp.Cr, ethanol-HCl induced gastric model was used which through variety of mechanisms stimulates ulcer including mucus exhaustion, mucosal damage, release of superoxide anion, hydro-peroxy free radicals, all these mechanisms prolonged the tissue oxidative stress and release of inflammatory mediators. Fp.Cr significantly decreased the surface ulceration as compared to that of control animals which received saline. In view of these results, methanol extract showed significant cytoprotection. The potential of Fp.Cr to produce anti-ulcer effect might be due to its CCB effect, as Ca^2+^ antagonist are well known to demonstrate such effects [21]. In pathophysiology of gastric ulcers, oxidative stresss plays a vital role. Antioxidant and nitric oxide free scaveneging activity has been reported by *Ficus palmata* [10] which may be responsible for its effectiveness as anti-ulcer agent.

Intestinal transit travelling, an important measurement factor that regulates the bioavailability of orally administered drugs/foods and determines the absorption intensity of luminal contents. More oftenly, timely oral administration of active charcoal, as marker, to experimental animals are useful to measure the intestinal transit rate. This experimental model is sensitive to agents that inhibit/stimulate intestinal peristalsis regulated by autonomic nervous system. This is the rationale for using this assay to investigate the influence of natural products on intestinal peristalsis [22]. In the small intestinal transit test, Fp.Cr produces suppression of the propulsion of charcoal marker at all test doses just like atropine sulphate a standard drug, that has been reported to have anticholinergic effect on intestinal transit [21]. A decrease in the motility of gut muscles increases the stay of substances in the intestine, thus allows better water absorption. This finding suggests that Fp.Cr has the ability to influence the peristaltic movement of intestine thereby indicating the presence of an anti-motility activity. It is therefore presumed that the reduction in the intestinal propulsive movement in the charcoal meal model may be due to antispasmodic properties of the Fp.Cr [22].

The observed therapeutic effects of *Ficus palmata* may be due to the presence of phytochemicals, tannins and flavonoids, as these phytocontinuents are well known for gastrointestinal effects. Anti-diarrheal, anti-secretory, anti-ulcer and anti-spasmodic activities may be due to flavonoids [23].

Molecular docking is an effective tool for evaluating the affinity of various protein targets that may possibly be associated with the pathophysiology of gastric disorders. The traditionally acclaimed use of *Ficus palmata* in the management of gastric related diseases has been supported with scientific evidence using virtual screening tool and different chemically-induced gastrointestinal models like castor oil induced diarrhea, castor oil induced enteropooling, and charcoal meal gastrointestinal motility. The use of computational methodologies has end up being fundamental component of drug discovery and development process therefore computational screening becomes broadly employed tool in identifying the potential of structural chemical compounds before initiation of wet lab research. Since early 90’s, docking has been observed to play a primary role for virtual screening of different chemical structures and it is still an extremely dynamic region to look forward [24]. Lead compounds are virtually screened through molecular docking that turns out to be time saving as well as economically affordable on premise of structural conformations [25].

In this study, Auto Dock Vina program was used through PyRx [26]. Through gradient optimization method, binding mode predictions are improved. E-value, Lower desolvation, Hydrogen bonding, pi–pi and other hydrophobic interactions imparts their influential effect in gastrointestinal diseases [27] which are contributing factors in increasing affinity with protein and stabilization of ligand-receptor complex. It has been found that psoralenoside showed excellent score of binding against α_1_ receptor with lowest E-value. This binding efficacy is greater than majority of the target proteins with better affinity as compared to other test compounds and standard drugs. Order of affinity of test compounds for α_1_ and M_1_ receptor was; psoralenoside > bergapten > psoralene > germanicol acetate. Compounds with higher affinity all together formed stronger pi–pi bonds, high number of hydrophobic interactions and polar hydrogen bonding against muscarinic M_1_ and α_1_ receptors. The order of affinity for ligands against muscranic M_3_ receptor was found as; bergapten > psoralene > psoralenoside > germanicol acetate. Order of affinity of test compounds for dopaminergic D_2_ receptor was found as; psoralenoside > bergapten > psoralene > germanicol acetate. Alongside hydrogen and hydrophobic interactions, different types of interactions, for example alky, pi-alky and vander waal interactions are appeared with high proclivity by test compounds. The affinity order of ligands against calmodulin was found as; bergapten > psoralene > psoralenoside > germanicol acetate. In addition, hydrogen bond is considered to be vital for complex of ligand with calmodulin. The affinity order for test compounds for voltage gated L-Type calcium channel was found as; bergapten > psoralene > psoralenoside > germanicol acetate. Order of affinity of test compounds for histaminergic H_1_ receptor was found to be: psoralene > bergapten > psoralenoside > germanicol acetate. Ligands are not engaged with making any solid interactions on stated restricting sites. Order of affinity of test compounds for H^+^/K^+^ ATPase receptor was found as; bergapten > psoralenoside > psoralene > germanicol acetate. Hydrogen and hydrophobic associations are observed to be essential but no such interactions of test compounds with stated restricting site were seen [28]. In this regard, SER 477 is considered as important and vital amino acid. The affinity order of ligands against histaminergic H_2_ receptor was found as; psoralenoside > psoralene > bergapten > germanicol acetate. Order of affinity of test compounds for mu-opioid receptor was found as: bergapten > psoralene > psoralenoside > germanicol acetate. Ligands having high restricting proclivity shaped interacts with TYR 272 and VAL 270.

## Conclusions

*Ficus palmata* exhibited anti-diarrheal, anti-secretary, anti-spasmodic, anti-motility and anti-ulcer effects. The plant constituents: psoralenoside and bergapten showed high binding affinities (E-value ≥ − 6.5 Kcal/mol) against histaminergic H_1_, calmodulin and voltage gated L-type calcium channels, while showed moderate affinities (E-value ≥7 Kcal/mol) against dopaminergic D_2_, adrenergic α_1,_ muscarinic M_3_, mu-opioid, whereas revealed lower affinities (E-value ≥9.5 Kcal/mol) vs. muscarinic M_1_, histaminergic H_2_ and H^+^/K^+^ ATPase pump. Germanicol acetate and psoralene exhibited weak affinities against aforementioned targets.

## Data Availability

The datasets used and/or analyzed during the current study are available from the corresponding author on reasonable request.

## Abbreviations

ACh: acetylcholine
ALA: alanine
ARG: arginine
ASN: asparagine
ASP: aspartic acid
cm: centimeter
CO_2_: carbon dioxide
CYS: cysteine
Fp.Cr: *Ficus palmata* Crude
g: gram
GLN: glutamine
GLU: glutamic acid
GLY: glycine
HIS: histidine
ILE: isoleucine
K^+^: Potassium
kg: kilogram
LYS: lysine
MET: methionine
mg: milligram
mL: mililitre
O_2_: oxygen
p.o.: per oral
PHE: phenylalanine
PRO: proline
SER: serine
THR: threonine
TRP: tryptophan
TYR: tyrosine
USc: ulcer surface area of control
USt: ulcer surface area of test drug group
VAL: valine
